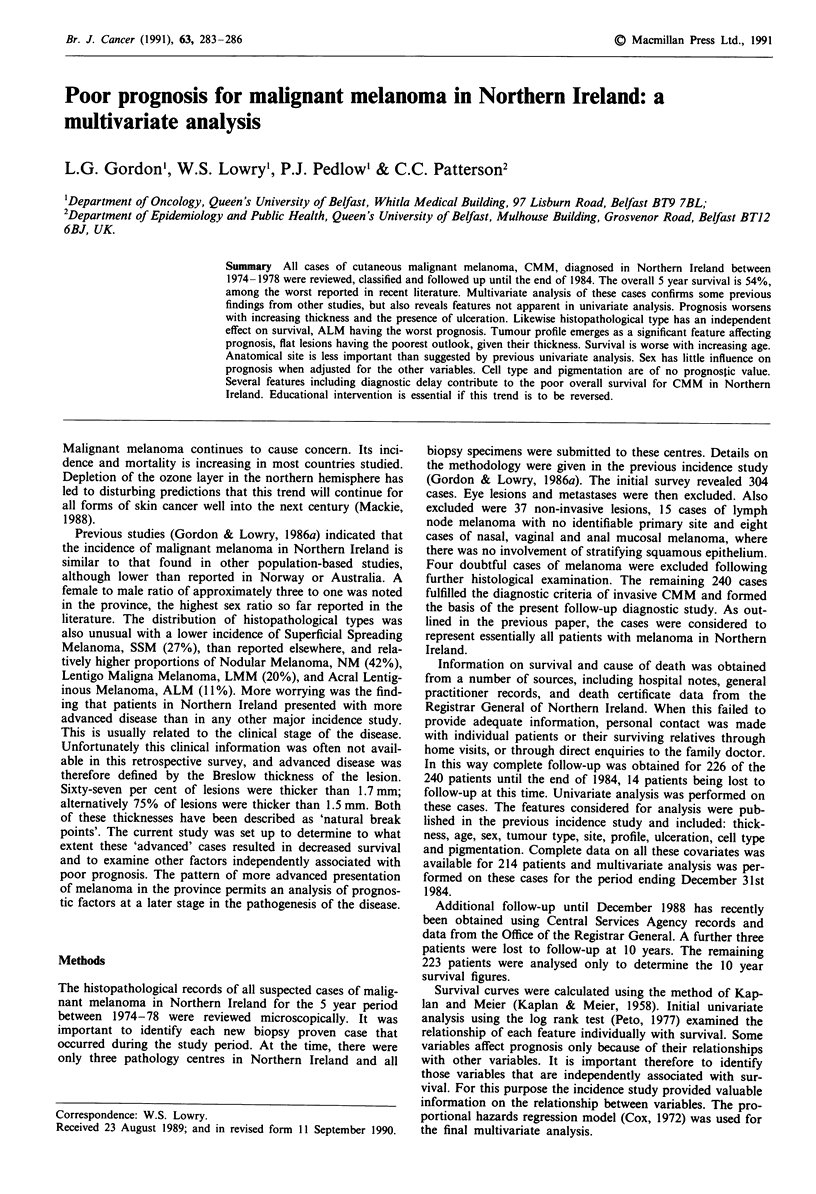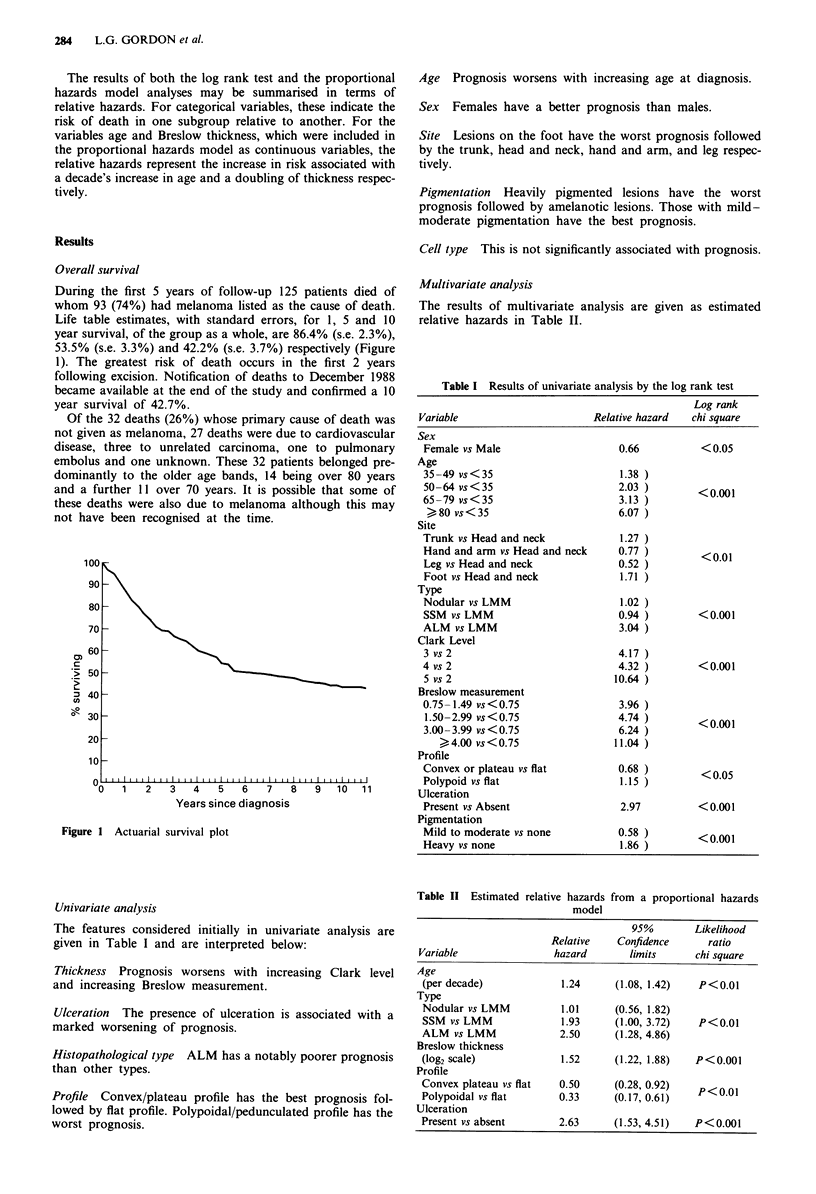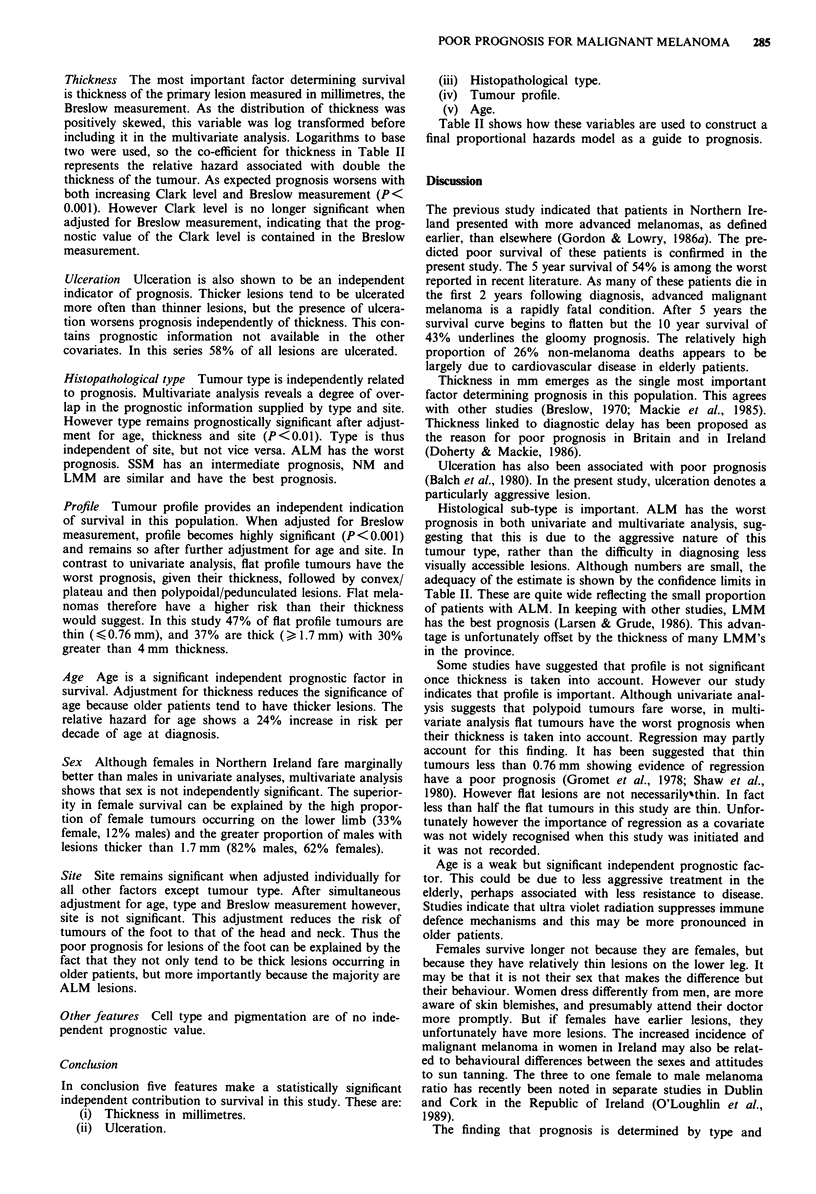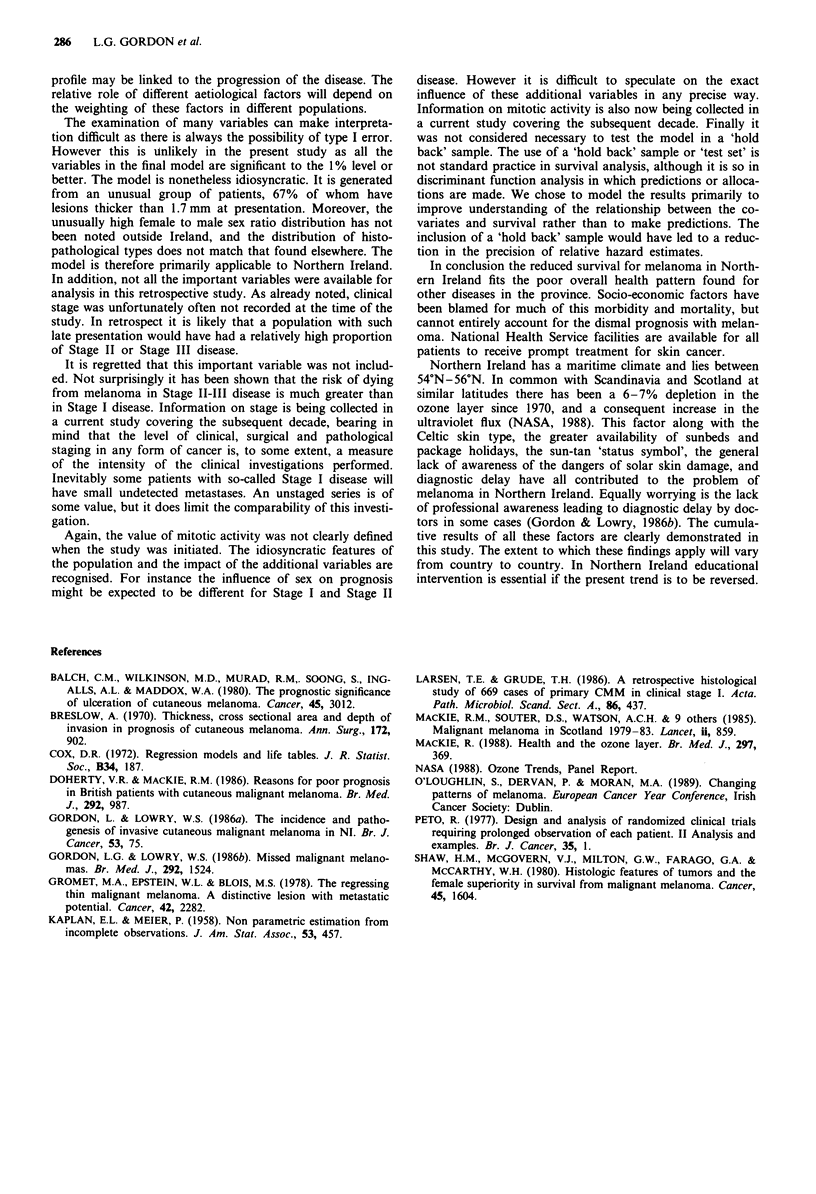# Poor prognosis for malignant melanoma in Northern Ireland: a multivariate analysis.

**DOI:** 10.1038/bjc.1991.66

**Published:** 1991-02

**Authors:** L. G. Gordon, W. S. Lowry, P. J. Pedlow, C. C. Patterson

**Affiliations:** Department of Oncology, Queen's University of Belfast, UK.

## Abstract

All cases of cutaneous malignant melanoma, CMM, diagnosed in Northern Ireland between 1974-1978 were reviewed, classified and followed up until the end of 1984. The overall 5 year survival is 54%, among the worst reported in recent literature. Multivariate analysis of these cases confirms some previous findings from other studies, but also reveals features not apparent in univariate analysis. Prognosis worsens with increasing thickness and the presence of ulceration. Likewise histopathological type has an independent effect on survival, ALM having the worst prognosis. Tumour profile emerges as a significant feature affecting prognosis, flat lesions having the poorest outlook, given their thickness. Survival is worse with increasing age. Anatomical site is less important than suggested by previous univariate analysis. Sex has little influence on prognosis when adjusted for the other variables. Cell type and pigmentation are of no prognostic value. Several features including diagnostic delay contribute to the poor overall survival for CMM in Northern Ireland. Educational intervention is essential if this trend is to be reversed.


Br. J. Cancer (1991), 63, 283-286 © Macmillan Press Ltd., 1991

### Poor prognosis for malignant melanoma in Northern Ireland: a multivariate analysis

L.G. Gordon[1], W.S. Lowry[1], P.J. Pedlow[1] & C.C. Patterson[2]

[1]*Department of Oncology, Queen's University of Belfast, Whitla Medical Building, 97 Lisburn Road, Belfast BT9 7BL;* [2]*Department of Epidemiology and Public Health, Queen's University of Belfast, Mulhouse Building, Grosvenor Road, Belfast BT12 6BJ, UK.*

**Summary** All cases of cutaneous malignant melanoma, CMM, diagnosed in Northern Ireland between 1974-1978 were reviewed, classified and followed up until the end of 1984. The overall 5 year survival is 54%, among the worst reported in recent literature. Multivariate analysis of these cases confirms some previous findings from other studies, but also reveals features not apparent in univariate analysis. Prognosis worsens with increasing thickness and the presence of ulceration. Likewise histopathological type has an independent effect on survival, ALM having the worst prognosis. Tumour profile emerges as a significant feature affecting prognosis, flat lesions having the poorest outlook, given their thickness. Survival is worse with increasing age. Anatomical site is less important than suggested by previous univariate analysis. Sex has little influence on prognosis when adjusted for the other variables. Cell type and pigmentation are of no prognostic value. Several features including diagnostic delay contribute to the poor overall survival for CMM in Northern Ireland. Educational intervention is essential if this trend is to be reversed.

Malignant melanoma continues to cause concern. Its incidence and mortality is increasing in most countries studied. Depletion of the ozone layer in the northern hemisphere has led to disturbing predictions that this trend will continue for all forms of skin cancer well into the next century (Mackie, 1988).

Previous studies (Gordon & Lowry, 1986*a*) indicated that the incidence of malignant melanoma in Northern Ireland is similar to that found in other population-based studies, although lower than reported in Norway or Australia. A female to male ratio of approximately three to one was noted in the province, the highest sex ratio so far reported in the literature. The distribution of histopathological types was also unusual with a lower incidence of Superficial Spreading Melanoma, SSM (27%), than reported elsewhere, and relatively higher proportions of Nodular Melanoma, NM (42%), Lentigo Maligna Melanoma, LMM (20%), and Acral Lentiginous Melanoma, ALM (11%). More worrying was the finding that patients in Northern Ireland presented with more advanced disease than in any other major incidence study. This is usually related to the clinical stage of the disease. Unfortunately this clinical information was often not available in this retrospective survey, and advanced disease was therefore defined by the Breslow thickness of the lesion. Sixty-seven per cent of lesions were thicker than 1.7 mm; alternatively 75% of lesions were thicker than 1.5 mm. Both of these thicknesses have been described as 'natural break points'. The current study was set up to determine to what extent these 'advanced' cases resulted in decreased survival and to examine other factors independently associated with poor prognosis. The pattern of more advanced presentation of melanoma in the province permits an analysis of prognostic factors at a later stage in the pathogenesis of the disease.

### Methods

The histopathological records of all suspected cases of malignant melanoma in Northern Ireland for the 5 year period between 1974-78 were reviewed microscopically. It was important to identify each new biopsy proven case that occurred during the study period. At the time, there were only three pathology centres in Northern Ireland and all biopsy specimens were submitted to these centres. Details on the methodology were given in the previous incidence study (Gordon & Lowry, 1986*a*). The initial survey revealed 304 cases. Eye lesions and metastases were then excluded. Also excluded were 37 non-invasive lesions, 15 cases of lymph node melanoma with no identifiable primary site and eight cases of nasal, vaginal and anal mucosal melanoma, where there was no involvement of stratifying squamous epithelium. Four doubtful cases of melanoma were excluded following further histological examination. The remaining 240 cases fulfilled the diagnostic criteria of invasive CMM and formed the basis of the present follow-up diagnostic study. As outlined in the previous paper, the cases were considered to represent essentially all patients with melanoma in Northern Ireland.

Information on survival and cause of death was obtained from a number of sources, including hospital notes, general practitioner records, and death certificate data from the Registrar General of Northern Ireland. When this failed to provide adequate information, personal contact was made with individual patients or their surviving relatives through home visits, or through direct enquiries to the family doctor. In this way complete follow-up was obtained for 226 of the 240 patients until the end of 1984, 14 patients being lost to follow-up at this time. Univariate analysis was performed on these cases. The features considered for analysis were published in the previous incidence study and included: thickness, age, sex, tumour type, site, profile, ulceration, cell type and pigmentation. Complete data on all these covariates was available for 214 patients and multivariate analysis was performed on these cases for the period ending December 31st 1984.

Additional follow-up until December 1988 has recently been obtained using Central Services Agency records and data from the Office of the Registrar General. A further three patients were lost to follow-up at 10 years. The remaining 223 patients were analysed only to determine the 10 year survival figures.

Survival curves were calculated using the method of Kaplan and Meier (Kaplan & Meier, 1958). Initial univariate analysis using the log rank test (Peto, 1977) examined the relationship of each feature individually with survival. Some variables affect prognosis only because of their relationships with other variables. It is important therefore to identify those variables that are independently associated with survival. For this purpose the incidence study provided valuable information on the relationship between variables. The proportional hazards regression model (Cox, 1972) was used for the final multivariate analysis.

Correspondence: W.S. Lowry.
Received 23 August 1989; and in revised form 11 September 1990.

The results of both the log rank test and the proportional hazards model analyses may be summarised in terms of relative hazards. For categorical variables, these indicate the risk of death in one subgroup relative to another. For the variables age and Breslow thickness, which were included in the proportional hazards model as continuous variables, the relative hazards represent the increase in risk associated with a decade's increase in age and a doubling of thickness respectively.

### Results

### *Overall survival*

During the first 5 years of follow-up 125 patients died of whom 93 (74%) had melanoma listed as the cause of death. Life table estimates, with standard errors, for 1, 5 and 10 year survival, of the group as a whole, are 86.4% (s.e. 2.3%), 53.5% (s.e. 3.3%) and 42.2% (s.e. 3.7%) respectively (Figure 1). The greatest risk of death occurs in the first 2 years following excision. Notification of deaths to December 1988 became available at the end of the study and confirmed a 10 year survival of 42.7%.

Of the 32 deaths (26%) whose primary cause of death was not given as melanoma, 27 deaths were due to cardiovascular disease, three to unrelated carcinoma, one to pulmonary embolus and one unknown. These 32 patients belonged predominantly to the older age bands, 14 being over 80 years and a further 11 over 70 years. It is possible that some of these deaths were also due to melanoma although this may not have been recognised at the time.

**Figure 1** Actuarial survival plot

### *Univariate analysis*

The features considered initially in univariate analysis are given in Table I and are interpreted below:

*Thickness* Prognosis worsens with increasing Clark level and increasing Breslow measurement.

*Ulceration* The presence of ulceration is associated with a marked worsening of prognosis.

*Histopathological type* ALM has a notably poorer prognosis than other types.

*Profile* Convex/plateau profile has the best prognosis followed by flat profile. Polypoidal/pedunculated profile has the worst prognosis.

*Age* Prognosis worsens with increasing age at diagnosis.

*Sex* Females have a better prognosis than males.

*Site* Lesions on the foot have the worst prognosis followed by the trunk, head and neck, hand and arm, and leg respectively.

*Pigmentation* Heavily pigmented lesions have the worst prognosis followed by amelanotic lesions. Those with mild–moderate pigmentation have the best prognosis.

*Cell type* This is not significantly associated with prognosis.

### *Multivariate analysis*

The results of multivariate analysis are given as estimated relative hazards in Table II.

**Table I** Results of univariate analysis by the log rank test

| *Variable* | *Relative hazard* | *Log rank chi square* |
|---|---|---|
| *Sex* | | |
| Female *vs* Male | 0.66 | <0.05 |
| Age | | |
| 35–49 *vs* <35 | 1.38 ) | |
| 50–64 *vs* <35 | 2.03 ) | <0.001 |
| 65–79 *vs* <35 | 3.13 ) | |
| ≥80 *vs* <35 | 6.07 ) | |
| Site | | |
| Trunk *vs* Head and neck | 1.27 ) | |
| Hand and arm *vs* Head and neck | 0.77 ) | <0.01 |
| Leg *vs* Head and neck | 0.52 ) | |
| Foot *vs* Head and neck | 1.71 ) | |
| Type | | |
| Nodular *vs* LMM | 1.02 ) | |
| SSM *vs* LMM | 0.94 ) | <0.001 |
| ALM *vs* LMM | 3.04 ) | |
| Clark Level | | |
| 3 *vs* 2 | 4.17 ) | |
| 4 *vs* 2 | 4.32 ) | <0.001 |
| 5 *vs* 2 | 10.64 ) | |
| Breslow measurement | | |
| 0.75–1.49 *vs* <0.75 | 3.96 ) | |
| 1.50–2.99 *vs* <0.75 | 4.74 ) | <0.001 |
| 3.00–3.99 *vs* <0.75 | 6.24 ) | |
| ≥4.00 *vs* <0.75 | 11.04 ) | |
| Profile | | |
| Convex or plateau *vs* flat | 0.68 ) | <0.05 |
| Polypoid *vs* flat | 1.15 ) | |
| Ulceration | | |
| Present *vs* Absent | 2.97 | <0.001 |
| Pigmentation | | |
| Mild to moderate *vs* none | 0.58 ) | <0.001 |
| Heavy *vs* none | 1.86 ) | |

**Table II** Estimated relative hazards from a proportional hazards model

| *Variable* | *Relative hazard* | *95% Confidence limits* | *Likelihood ratio chi square* |
|---|---|---|---|
| *Age* | | | |
| (per decade) | 1.24 | (1.08, 1.42) | *P*<0.01 |
| Type | | | |
| Nodular *vs* LMM | 1.01 | (0.56, 1.82) | |
| SSM *vs* LMM | 1.93 | (1.00, 3.72) | *P*<0.01 |
| ALM *vs* LMM | 2.50 | (1.28, 4.86) | |
| Breslow thickness | | | |
| ($\log_2$ scale) | 1.52 | (1.22, 1.88) | *P*<0.001 |
| Profile | | | |
| Convex plateau *vs* flat | 0.50 | (0.28, 0.92) | *P*<0.01 |
| Polypoidal *vs* flat | 0.33 | (0.17, 0.61) | |
| Ulceration | | | |
| Present *vs* absent | 2.63 | (1.53, 4.51) | *P*<0.001 |

*Thickness* The most important factor determining survival is thickness of the primary lesion measured in millimetres, the Breslow measurement. As the distribution of thickness was positively skewed, this variable was log transformed before including it in the multivariate analysis. Logarithms to base two were used, so the co-efficient for thickness in Table II represents the relative hazard associated with double the thickness of the tumour. As expected prognosis worsens with both increasing Clark level and Breslow measurement ($P <$ 0.001). However Clark level is no longer significant when adjusted for Breslow measurement, indicating that the prognostic value of the Clark level is contained in the Breslow measurement.

*Ulceration* Ulceration is also shown to be an independent indicator of prognosis. Thicker lesions tend to be ulcerated more often than thinner lesions, but the presence of ulceration worsens prognosis independently of thickness. This contains prognostic information not available in the other covariates. In this series 58% of all lesions are ulcerated.

*Histopathological type* Tumour type is independently related to prognosis. Multivariate analysis reveals a degree of overlap in the prognostic information supplied by type and site. However type remains prognostically significant after adjustment for age, thickness and site ($P < 0.01$). Type is thus independent of site, but not vice versa. ALM has the worst prognosis. SSM has an intermediate prognosis, NM and LMM are similar and have the best prognosis.

*Profile* Tumour profile provides an independent indication of survival in this population. When adjusted for Breslow measurement, profile becomes highly significant ($P < 0.001$) and remains so after further adjustment for age and site. In contrast to univariate analysis, flat profile tumours have the worst prognosis, given their thickness, followed by convex/ plateau and then polypoidal/pedunculated lesions. Flat melanomas therefore have a higher risk than their thickness would suggest. In this study 47% of flat profile tumours are thin ($\leqslant 0.76$ mm), and 37% are thick ($\geqslant 1.7$ mm) with 30% greater than 4 mm thickness.

*Age* Age is a significant independent prognostic factor in survival. Adjustment for thickness reduces the significance of age because older patients tend to have thicker lesions. The relative hazard for age shows a 24% increase in risk per decade of age at diagnosis.

*Sex* Although females in Northern Ireland fare marginally better than males in univariate analyses, multivariate analysis shows that sex is not independently significant. The superiority in female survival can be explained by the high proportion of female tumours occurring on the lower limb (33% female, 12% males) and the greater proportion of males with lesions thicker than 1.7 mm (82% males, 62% females).

*Site* Site remains significant when adjusted individually for all other factors except tumour type. After simultaneous adjustment for age, type and Breslow measurement however, site is not significant. This adjustment reduces the risk of tumours of the foot to that of the head and neck. Thus the poor prognosis for lesions of the foot can be explained by the fact that they not only tend to be thick lesions occurring in older patients, but more importantly because the majority are ALM lesions.

*Other features* Cell type and pigmentation are of no independent prognostic value.

*Conclusion*

In conclusion five features make a statistically significant independent contribution to survival in this study. These are:
(i) Thickness in millimetres.
(ii) Ulceration.
(iii) Histopathological type.
(iv) Tumour profile.
(v) Age.

Table II shows how these variables are used to construct a final proportional hazards model as a guide to prognosis.

### Discussion

The previous study indicated that patients in Northern Ireland presented with more advanced melanomas, as defined earlier, than elsewhere (Gordon & Lowry, 1986*a*). The predicted poor survival of these patients is confirmed in the present study. The 5 year survival of 54% is among the worst reported in recent literature. As many of these patients die in the first 2 years following diagnosis, advanced malignant melanoma is a rapidly fatal condition. After 5 years the survival curve begins to flatten but the 10 year survival of 43% underlines the gloomy prognosis. The relatively high proportion of 26% non-melanoma deaths appears to be largely due to cardiovascular disease in elderly patients.

Thickness in mm emerges as the single most important factor determining prognosis in this population. This agrees with other studies (Breslow, 1970; Mackie *et al.*, 1985). Thickness linked to diagnostic delay has been proposed as the reason for poor prognosis in Britain and in Ireland (Doherty & Mackie, 1986).

Ulceration has also been associated with poor prognosis (Balch *et al.*, 1980). In the present study, ulceration denotes a particularly aggressive lesion.

Histological sub-type is important. ALM has the worst prognosis in both univariate and multivariate analysis, suggesting that this is due to the aggressive nature of this tumour type, rather than the difficulty in diagnosing less visually accessible lesions. Although numbers are small, the adequacy of the estimate is shown by the confidence limits in Table II. These are quite wide reflecting the small proportion of patients with ALM. In keeping with other studies, LMM has the best prognosis (Larsen & Grude, 1986). This advantage is unfortunately offset by the thickness of many LMM's in the province.

Some studies have suggested that profile is not significant once thickness is taken into account. However our study indicates that profile is important. Although univariate analysis suggests that polypoid tumours fare worse, in multivariate analysis flat tumours have the worst prognosis when their thickness is taken into account. Regression may partly account for this finding. It has been suggested that thin tumours less than 0.76 mm showing evidence of regression have a poor prognosis (Gromet *et al.*, 1978; Shaw *et al.*, 1980). However flat lesions are not necessarily thin. In fact less than half the flat tumours in this study are thin. Unfortunately however the importance of regression as a covariate was not widely recognised when this study was initiated and it was not recorded.

Age is a weak but significant independent prognostic factor. This could be due to less aggressive treatment in the elderly, perhaps associated with less resistance to disease. Studies indicate that ultra violet radiation suppresses immune defence mechanisms and this may be more pronounced in older patients.

Females survive longer not because they are females, but because they have relatively thin lesions on the lower leg. It may be that it is not their sex that makes the difference but their behaviour. Women dress differently from men, are more aware of skin blemishes, and presumably attend their doctor more promptly. But if females have earlier lesions, they unfortunately have more lesions. The increased incidence of malignant melanoma in women in Ireland may also be related to behavioural differences between the sexes and attitudes to sun tanning. The three to one female to male melanoma ratio has recently been noted in separate studies in Dublin and Cork in the Republic of Ireland (O'Loughlin *et al.*, 1989).

The finding that prognosis is determined by type and profile may be linked to the progression of the disease. The relative role of different aetiological factors will depend on the weighting of these factors in different populations.

The examination of many variables can make interpretation difficult as there is always the possibility of type I error. However this is unlikely in the present study as all the variables in the final model are significant to the 1% level or better. The model is nonetheless idiosyncratic. It is generated from an unusual group of patients, 67% of whom have lesions thicker than 1.7 mm at presentation. Moreover, the unusually high female to male sex ratio distribution has not been noted outside Ireland, and the distribution of histopathological types does not match that found elsewhere. The model is therefore primarily applicable to Northern Ireland. In addition, not all the important variables were available for analysis in this retrospective study. As already noted, clinical stage was unfortunately often not recorded at the time of the study. In retrospect it is likely that a population with such late presentation would have had a relatively high proportion of Stage II or Stage III disease.

It is regretted that this important variable was not included. Not surprisingly it has been shown that the risk of dying from melanoma in Stage II-III disease is much greater than in Stage I disease. Information on stage is being collected in a current study covering the subsequent decade, bearing in mind that the level of clinical, surgical and pathological staging in any form of cancer is, to some extent, a measure of the intensity of the clinical investigations performed. Inevitably some patients with so-called Stage I disease will have small undetected metastases. An unstaged series is of some value, but it does limit the comparability of this investigation.

Again, the value of mitotic activity was not clearly defined when the study was initiated. The idiosyncratic features of the population and the impact of the additional variables are recognised. For instance the influence of sex on prognosis might be expected to be different for Stage I and Stage II disease. However it is difficult to speculate on the exact influence of these additional variables in any precise way. Information on mitotic activity is also now being collected in a current study covering the subsequent decade. Finally it was not considered necessary to test the model in a 'hold back' sample. The use of a 'hold back' sample or 'test set' is not standard practice in survival analysis, although it is so in discriminant function analysis in which predictions or allocations are made. We chose to model the results primarily to improve understanding of the relationship between the co-variates and survival rather than to make predictions. The inclusion of a 'hold back' sample would have led to a reduction in the precision of relative hazard estimates.

In conclusion the reduced survival for melanoma in Northern Ireland fits the poor overall health pattern found for other diseases in the province. Socio-economic factors have been blamed for much of this morbidity and mortality, but cannot entirely account for the dismal prognosis with melanoma. National Health Service facilities are available for all patients to receive prompt treatment for skin cancer.

Northern Ireland has a maritime climate and lies between 54°N–56°N. In common with Scandinavia and Scotland at similar latitudes there has been a 6–7% depletion in the ozone layer since 1970, and a consequent increase in the ultraviolet flux (NASA, 1988). This factor along with the Celtic skin type, the greater availability of sunbeds and package holidays, the sun-tan 'status symbol', the general lack of awareness of the dangers of solar skin damage, and diagnostic delay have all contributed to the problem of melanoma in Northern Ireland. Equally worrying is the lack of professional awareness leading to diagnostic delay by doctors in some cases (Gordon & Lowry, 1986*b*). The cumulative results of all these factors are clearly demonstrated in this study. The extent to which these findings apply will vary from country to country. In Northern Ireland educational intervention is essential if the present trend is to be reversed.

### References

BALCH, C.M., WILKINSON, M.D., MURAD, R.M,. SOONG, S., INGALLS, A.L. & MADDOX, W.A. (1980). The prognostic significance of ulceration of cutaneous melanoma. *Cancer*, **45**, 3012.

BRESLOW, A. (1970). Thickness, cross sectional area and depth of invasion in prognosis of cutaneous melanoma. *Ann. Surg.*, **172**, 902.

COX, D.R. (1972). Regression models and life tables. *J. R. Statist. Soc.*, **B34**, 187.

DOHERTY, V.R. & MACKIE, R.M. (1986). Reasons for poor prognosis in British patients with cutaneous malignant melanoma. *Br. Med. J.*, **292**, 987.

GORDON, L. & LOWRY, W.S. (1986*a*). The incidence and pathogenesis of invasive cutaneous malignant melanoma in NI. *Br. J. Cancer*, **53**, 75.

GORDON, L.G. & LOWRY, W.S. (1986*b*). Missed malignant melanomas. *Br. Med. J.*, **292**, 1524.

GROMET, M.A., EPSTEIN, W.L. & BLOIS, M.S. (1978). The regressing thin malignant melanoma. A distinctive lesion with metastatic potential. *Cancer*, **42**, 2282.

KAPLAN, E.L. & MEIER, P. (1958). Non parametric estimation from incomplete observations. *J. Am. Stat. Assoc.*, **53**, 457.

LARSEN, T.E. & GRUDE, T.H. (1986). A retrospective histological study of 669 cases of primary CMM in clinical stage I. *Acta. Path. Microbiol. Scand. Sect. A.*, **86**, 437.

MACKIE, R.M., SOUTER, D.S., WATSON, A.C.H. & 9 others (1985). Malignant melanoma in Scotland 1979–83. *Lancet*, **ii**, 859.

MACKIE, R. (1988). Health and the ozone layer. *Br. Med. J.*, **297**, 369.

NASA (1988). Ozone Trends, Panel Report.

O'LOUGHLIN, S., DERVAN, P. & MORAN, M.A. (1989). Changing patterns of melanoma. *European Cancer Year Conference*, Irish Cancer Society: Dublin.

PETO, R. (1977). Design and analysis of randomized clinical trials requiring prolonged observation of each patient. II Analysis and examples. *Br. J. Cancer*, **35**, 1.

SHAW, H.M., MCGOVERN, V.J., MILTON, G.W., FARAGO, G.A. & MCCARTHY, W.H. (1980). Histologic features of tumors and the female superiority in survival from malignant melanoma. *Cancer*, **45**, 1604.